# The efficacy of contrast-enhanced harmonic endoscopic ultrasonography in diagnosing gallbladder cancer

**DOI:** 10.1038/srep25848

**Published:** 2016-05-10

**Authors:** Mitsuru Sugimoto, Tadayuki Takagi, Naoki Konno, Rei Suzuki, Hiroyuki Asama, Takuto Hikichi, Ko Watanabe, Yuichi Waragai, Hitomi Kikuchi, Mika Takasumi, Hiromasa Ohira

**Affiliations:** 1 Department of Gastroenterology and Rheumatology, Fukushima Medical University, School of Medicine, Fukushima, Japan; 2 Department of Endoscopy, Fukushima Medical University Hospital, Fukushima, Japan

## Abstract

The aim of this study was to review the efficacy of contrast-enhanced harmonic endoscopic ultrasonography (CH-EUS) in diagnosing gallbladder (GB)-protruded lesions. Thirty-eight patients underwent CH-EUS for the diagnosis of GB-protruded lesions. Twenty-four patients whose major axes of their largest lesions were longer than 10 mm were recruited. The ability of CH-EUS to diagnose malignant or benign lesions was reviewed. We treated lesions with brindled enhanced patterns as malignant and those with uniformly enhanced or unenhanced patterns as benign. Furthermore, three gastroenterologists who were not familiar with pancreaticobiliary EUS compared the diagnostic abilities of CH-EUS and conventional EUS using photographs. The sensitivity, specificity, and malignant accuracy of CH-EUS were 100, 94.4, and 95.8%, respectively. The number of lesions that presented with enhanced patterns was significantly different between the malignant lesions and the benign lesions (*P* < 0.001). In the comparison of diagnostic abilities between CH-EUS and conventional EUS by the three gastroenterologists, CH-EUS was significantly superior to conventional EUS in terms of sensitivity, specificity, and accuracy (76.1 vs. 42.9%, *P* = 0.029; 66.7 vs. 39.2%, *P* = 0.005; and 69.4 vs. 40.3%, *P* < 0.001; respectively). In conclusion, CH-EUS was useful for diagnosing malignant and benign GB-protruded lesions.

Distinguishing organs on percutaneous abdominal ultrasonography (US) can be difficult, particularly if a patient has a large amount of subcutaneous fat or intestinal gas. Endoscopic ultrasonography (EUS) from the lumen of the stomach or duodenum can be used to visualise organs more clearly and completely because of its high spatial resolution. In addition, EUS rarely has blind spots. Because of the high detection capability of EUS, it is preferred over US or computerised tomography (CT) for investigating pancreaticobiliary diseases^1^,^2^.

Several investigators have reported on the diagnosis of gallbladder (GB)-protruded lesions. In 1999, Sugiyama *et al*.^3^ reported that EUS was superior to US in distinguishing among GB neoplastic lesions (adenoma or adenocarcinoma), cholesterol polyps (CPs), and adenomyomatosis (ADM). Furthermore, in 2000, Sugiyama *et al*.^4^ reported that the diagnostic ability of EUS (97%) was superior to that of US (76%) for GB-protruded lesions. Kimura *et al*.^5^ reported that the evaluation of lesion contours by EUS was useful for diagnosing GB neoplastic lesions, and Sadamoto *et al*. reported that scoring using EUS was useful for diagnosing GB neoplastic lesions^6^. However, these reports did not intend to convey that EUS can be used to diagnose only GB adenocarcinomas. Jang *et al*. also reported that the diagnostic ability of EUS is equivalent to that of high-resolution US^7^.

The efficacy of contrast-enhanced harmonic endoscopic ultrasonography (CH-EUS) in diagnosing GB malignant lesions has also been reported. In 1997 and 1998, Hirooka *et al*.^8^,^9^ reported that visualization of GB adenocarcinoma was enhanced by the contrast medium Albunex (Shionogi, Osaka, Japan). In addition, Choi *et al*.^10^ reported the efficacy of CH-EUS using the contrast medium SonoVue (Bracco International BV, Amsterdam, Netherlands). In CH-EUS, irregular vessels or perfusion defects were observed in GB malignant lesions^10^. In addition, Park *et al*.^11^ reported that 80% of GB adenocarcinomas were heterogeneously enhanced and 75% of GB adenomas were homogenously enhanced using SonoVue in the same manner. However, many CPs or ADMs show heterogeneously enhanced patterns, which makes it difficult to diagnose GB malignant lesions.

In Japan, the second-generation contrast medium Sonazoid (perflubutane; Daiichi-Sankyo, Tokyo, Japan) is used for EUS in pancreaticobiliary or other abdominal diseases^12^,^13^,^14^,^15^,^16^,^17^,^18^,^19^,^20^,^21^. It is suitable for EUS because it resonates at a low acoustic pressure^18^,^19^,^20^,^21^. Few bubbles are broken during examination; therefore, long observation periods are possible. Imazu *et al*. reported that inhomogeneously enhanced patterns were observed in CH-EUS with the use of Sonazoid, indicating malignant GB wall thickening^14^.

We performed CH-EUS using Sonazoid to diagnose malignant GB-protruded lesions. The purpose of this study was to retrospectively review the efficacy of CH-EUS in diagnosing malignant and benign large GB lesions.

## Results

### Patient characteristics and diagnostic ability of CH-EUS

**Figure 1:**
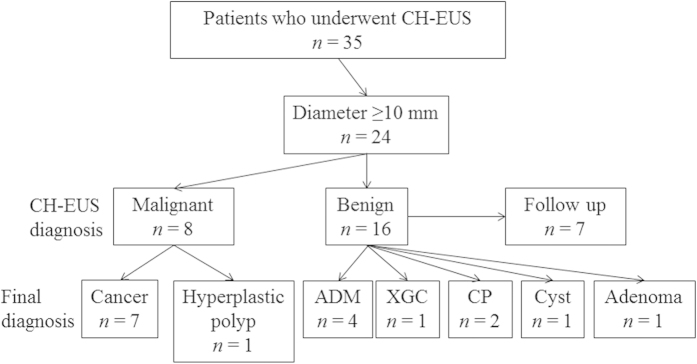
Diagnoses by contrast-enhanced harmonic endoscopic ultrasonography. *ADM* adenomyomatosis, *CH-EUS* contrast-enhanced harmonic endoscopic ultrasonography, *CP* cholesterol polyp, and *XGC* xanthogranulomatous cholecystisis.

The average age of the patients was 61.8 ± 15.1 years (range, 25–82 years). Eight men and 16 women participated in this study. The average long axis of the lesions investigated was 17.6 ± 7.8 mm (range, 10–37 mm). Based on CH-EUS, eight patients were diagnosed with malignant lesions (brindled enhanced pattern), and 16 patients were diagnosed with benign lesions (uniformly enhanced pattern or unenhanced pattern). All eight patients who were diagnosed with malignant lesions by CH-EUS underwent surgery. The pathological diagnoses were seven GB cancers and one hyperplastic polyp (Fig. 1). Nine of the 16 patients diagnosed with benign lesions by CH-EUS underwent surgery. The pathological diagnoses of these lesions included four ADM, one xanthogranulomatous cholecystisis (XGC), two CP, one GB cyst, and one adenoma. The XGC, CP, and adenoma cases were uniformly enhanced, and the ADM cases were also uniformly enhanced except for the Rokitansky–Aschoff sinuses. The GB cyst was unenhanced by CH-EUS. An additional seven patients did not wish to undergo surgery and therefore were followed up by US, EUS, CT, or magnetic resonance imaging. These patients were subsequently diagnosed with benign tumours. The accuracy of CH-EUS for diagnosing malignant lesions was 95.8% (23/24 patients). The sensitivity of CH-EUS was 100% (7/7), and the specificity of CH-EUS was 94.1% (16/17). The number of lesions with enhanced patterns indicative of GB cancer and the number of lesions with benign GB polyps were significantly different (*P* < 0.001) (Table 1).

### The comparison of CH-EUS and conventional EUS by three gastroenterologists (inexperienced endosonographers)

The results of the comparison between conventional EUS and CH-EUS are described as follows (conventional EUS vs. CH-EUS) (Table 2). The sensitivity, specificity, and accuracy of gastroenterologist A using CH-EUS compared to the same individual using conventional EUS were 57.1 (4/7) vs. 85.7% (6/7), *P* = 0.279; 29.4 (5/17) vs. 47.1% (8/17), *P* = 0.241; and 37.5 (9/24) vs. 58.3% (14/24), *P* = 0.149; respectively. The sensitivity, specificity, and accuracy of gastroenterologist B using CH-EUS compared to the same individual using conventional EUS were 57.1 (4/7) vs. 71.4% (5/7), *P* = 0.500; 52.9 (9/17) vs. 76.5% (13/17), *P* = 0.140; and 54.0 (13/24) vs. 75.0% (18/24), *P* = 0.131; respectively. The sensitivity, specificity, and accuracy of gastroenterologist C using CH-EUS compared to the same individual using conventional EUS were 14.3 (1/7) vs. 71.4% (5/7), *P* = 0.051; 35.3 (6/17) vs. 76.5% (13/17), *P* = 0.018; and 29.2 (7/24) vs. 75.0% (18/24), *P* < 0.001; respectively. The overall sensitivity, specificity, and accuracy of all three gastroenterologists using CH-EUS compared to the same individual using conventional EUS were 42.9 (9/21) vs. 76.1% (16/21), *P* = 0.029; 39.2 (20/51) vs. 66.7% (34/51), *P* = 0.005; and 40.3 (29/72) vs. 69.4% (50/72), *P* < 0.001; respectively. Gastroenterologist C’s specificity and accuracy using CH-EUS were statistically superior to those obtained using conventional EUS. Considering the results from all three gastroenterologists, the sensitivity, specificity, and accuracy of CH-EUS were statistically superior to those obtained with conventional EUS.

## Discussion

In this study, we reviewed the efficacy of CH-EUS using Sonazoid for diagnosing malignant large GB-protruded lesions. GB cancers show a brindled enhanced pattern, whereas benign GB tumours show uniformly enhanced or unenhanced patterns. The accuracy of CH-EUS in diagnosing malignant lesions was excellent at 95.8% (23/24 patients). The enhanced patterns of GB cancers and benign GB polyps were significantly different. In another investigation by three inexperienced endosonographers, the sensitivity, specificity, and accuracy of CH-EUS were better than those obtained with conventional EUS for diagnosing malignant GB-protruded lesions. Furthermore, the diagnostic ability of CH-EUS was statistically superior to that of conventional EUS in terms of specificity and accuracy for gastroenterologist C and sensitivity, specificity, and accuracy for all three gastroenterologists overall.

As mentioned in the Introduction, many investigators have reported on the efficacy of EUS in diagnosing GB-protruded lesions. Special features of GB neoplastic lesions, based on EUS observations, included morphological features such as smooth margins, internal echo heterogeneity, and flatness^3^,^4^ . A study investigating only the margins reported that GB neoplastic lesions were smooth and nodal^5^. This investigation reported the accuracy of EUS in detecting neoplastic polyps; however, only adenocarcinomas were not diagnosed. In actuality, many GB cancers are not flat; therefore, a diagnosis based on shape has its limitations.

By contrast, the usefulness of CH-EUS for diagnosing GB carcinoma has been reported in the past^8^,^9^,^10^,^11^,^14^. In two reports on CH-EUS using the second-generation contrast agent SonoVue (Bracco International BV), many GB carcinomas were heterogeneously enhanced^10^,^11^. Imazu *et al*. reported that homogeneously enhanced patterns in CH-EUS using Sonazoid were the malignant predictive factors of GB wall thickening^14^. In this study, all GB cancers showed a brindled enhanced pattern; however, 94.1% (16/17) of benign GB tumours showed uniformly enhanced or unenhanced patterns. The brindled enhanced pattern in CH-EUS using Sonazoid was also extremely useful for the diagnosis of malignant large GB-protruded lesions. In addition, diagnoses by enhanced patterns were easier than diagnoses by shape because of the results obtained from the three gastroenterologists who were not experienced in the use of CH-EUS. However, Park *et al*. reported that CPs are also enhanced heterogeneously^11^. CPs were uniformly enhanced in this study. It remains unknown whether the cause of this difference was a result of differences between the contrast medias SonoVue and Sonazoid or because of the definition of brindled enhancement as a low echoic area only after the contrast agent injection in this study.

One reason for the low echoic areas observed after contrast media administration is reported as follows. Choi *et al*.^10^ reported that GB carcinoma was enhanced heterogeneously because of a desmoplastic reaction in tumours. Fibroblasts in the stroma of cancer tissues are called “cancer-associated fibroblasts.” Fibroblasts induce the reconstitution of the extracellular matrix and cause fibrosis or a stromal reaction (i.e., a desmoplastic reaction)^22^ . Moreover, fibrosis was reported to be the cause of an unenhanced area in CH-EUS for pancreatic cancer^23^.

This study has some limitations. First, this was a retrospective study that involved a small number of patients at one institution. It is necessary to accumulate more patients or to perform a large-scale prospective study. Second, the recognition of enhanced patterns was based on a subjective index. The efficacy of the time intensity curve based on CH-EUS has been reported in solid pancreatic tumour diagnoses^24^. It is hoped that the time intensity curve based on CH-EUS can be applied to GB lesions. Third, concerning the additional diagnostic ability of CH-EUS, only doctors who do not perform pancreaticobiliary EUS professionally review malignancy using still images. All pancreaticobiliary specialists performed CH-EUS in this study; therefore, they did not participate in the test comparing the diagnostic abilities of CH-EUS and conventional EUS. However, as mentioned previously, the accuracy of CH-EUS to diagnose malignant or benign legions as assessed by the specialists was good. If three unexperienced endosonographers would have reviewed the lesions by video, the enhanced effect would have been observed more precisely and provided better and more promising results.

In conclusion, the enhanced pattern of CH-EUS was useful for the diagnosis of malignant or benign large GB-protruded lesions.

## Patients and Methods

### Study design

This study was a retrospective study. To evaluate the efficacy of CH-EUS in diagnosing malignant GB-protruded lesions, we examined the diagnostic ability of CH-EUS in distinguishing between benign and malignant lesions. In addition, three gastroenterologists diagnosed benign or malignant GB lesions by observing conventional EUS images and CH-EUS images, and the diagnostic abilities were compared between CH-EUS and conventional EUS. This study was approved by the ethics committee of Fukushima Medical University (Fukushima, Japan, authorization no. 2399). The methods were performed in accordance with the approved guidelines.

### Patients

Between February 2012 and March 2015, 38 patients underwent CH-EUS for the diagnosis of GB-protruded lesions. The patients were not required to give informed consent in this study because the analysis used anonymised data obtained after each patient agreed to medical examination by written consent. Of these, 24 patients in whom the major axis of the largest lesions was longer than 10 mm were recruited. GB lesions larger than 10 mm have been regarded as operable in past studies^25^,^26^.

### Contrast-enhanced harmonic endoscopic ultrasonography

The endoscope and ultrasonic equipment used in this study were the GF-UCT260 ultrasound gastrovideoscope (Olympus Medical System, Tokyo, Japan), the ProSound α-10 ultrasound system (Aloka, Tokyo, Japan), or the EU-ME2 ultrasound processor (Olympus Medical system, Tokyo, Japan). All the patients were sufficiently sedated with midazolam before the endoscope was inserted. After the target lesions were visualized on the monitor in the B mode and in the extended pure harmonic detection (ExPHD) mode, 0.015 mL/kg of the contrast medium (16 μg of Sonazoid [Daiichi-Sankyo] and 2 mL of distilled water) was injected. After, the GB-protruded lesions were evaluated in the arterial and early venous phases, which occurred approximately 90 seconds after the lesions were enhanced^13^,^27^. These lesions were diagnosed by the enhanced pattern. The diagnoses were performed by four doctors (T.T., R.S, M.S., and N.K.) who had experienced not less than 500 cases of pancreaticobiliary EUS.

### Diagnosis of malignancy

**Figure 2:**
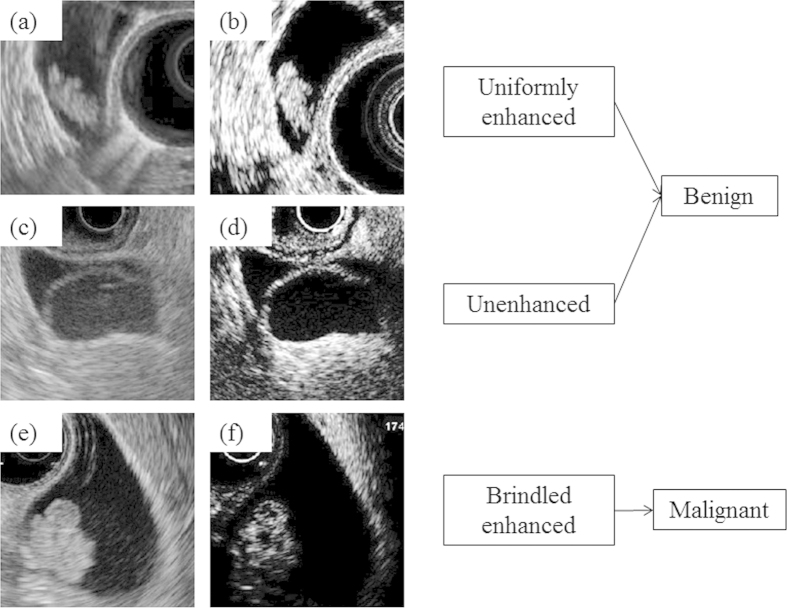
Enhanced pattern of gallbladder (GB) lesions in contrast-enhanced harmonic endoscopic ultrasonography (CH-EUS). Typical enhanced patterns were as published. In (**a**) the B mode image and (**b**) the extended pure harmonic detection (ExPHD) mode image, the lesion is uniformly enhanced after the contrast agent injection. The lesion was diagnosed as benign in CH-EUS. After surgery, the lesion was pathologically diagnosed as an adenoma. In (**c**) the B mode image and (**d**) the ExPHD mode image, the lesion was not enhanced after the contrast agent injection. Therefore, it was diagnosed as a benign lesion in CH-EUS. It was pathologically diagnosed as a GB cyst. In (**e**) the B mode image and (**f**) the ExPHD mode image after the contrast agent injection, several hypoechoic spots were observed, and the lesion was enhanced heterogeneously. The lesion was diagnosed as GB carcinoma in CH-EUS. The resected specimen was pathologically diagnosed as GB carcinoma.

The target lesions were diagnosed by the enhanced pattern. Uniformly enhanced lesions were diagnosed as benign lesions, such as an adenomas or CPs (Fig. 2a,b). Unenhanced lesions were also diagnosed as benign lesions (Fig. 2c,d). The lesions with brindled enhanced patterns were diagnosed as malignant (Fig. 2e,f). To exclude the Rokitansky–Aschoff sinuses in ADMs, CPs, or cysts, a spotty low echoic area that was evident only after contrast enhancement was defined as a brindled enhanced area^10^. The final diagnoses were based on the pathology results. The patients who did not undergo surgery because of a benign diagnosis in CH-EUS were finally diagnosed after a follow-up period longer than 6 months (the average observation period was 18.4 months). However, three gastroenterologists (A.H., T.M., and W.Y.) diagnosed 24 lesions as malignant or benign based on the B mode and ExPHD mode photographs from each case. They experienced no more than 100 cases of pancreaticobiliary EUS and not less than 500 abdominal US cases. They did not have any experience with CH-EUS. In the B mode, the diagnoses were performed as follows: nodular or smooth surfaced heterogeneously echogenic sessile masses were diagnosed as malignant polyps, granular surfaced homogeneously echogenic pedunculated masses were diagnosed as benign polyps, and smooth surfaced sessile masses with multiple anechoic cysts were diagnosed as ADMs. These methods are taken from past reports^4^,^5^,^28^. In CH-EUS, the diagnoses were performed as described.

### Statistical analysis

Analyses of the comparison of enhanced patterns between GB cancers and benign GB lesions, the comparison of sensitivity and specificity among gastroenterologists A, B, and C between CH-EUS and conventional EUS, and the comparison of sensitivity of all three gastroenterologists between CH-EUS and conventional EUS were performed by Fisher’s exact probability test. Analyses of the accuracy comparison for each gastroenterologist between CH-EUS and conventional EUS and the comparison of specificity and accuracy for all three gastroenterologists overall between CH-EUS and conventional EUS were performed by the Chi-square for independence test. *P* < 0.05 was considered statistically significant in all statistical analyses. All the tests were performed using Statcel 3 (OMS edition, Saitama, Japan).

## Additional Information

**How to cite this article**: Sugimoto, M. *et al*. The efficacy of contrast-enhanced harmonic endoscopic ultrasonography in diagnosing gallbladder cancer. *Sci. Rep.* **6**, 25848; doi: 10.1038/srep25848 (2016).

## Figures and Tables

**Table 1:** The results of enhanced patterns in GB lesions.

	GB cancer	Benign GB lesion	*P* value
n	7	17	<0.001
Brindled enhanced	7	1	
Uniformly enhanced or unenhanced	0	16	

*GB* gallbladder.

**Table 2:** Diagnostic ability of conventional endoscopic ultrasonography versus contrast-enhanced harmonic endoscopic ultrasonography by three gastroenterologists

	Conventional EUS	CH-EUS	*P*value
Gastroenterologist A			
Sensitivity (%)	57.1 (4/7)	85.7 (6/7)	0.279
Specificity (%)	29.4 (5/17)	47.1 (8/17)	0.241
Accuracy (%)	37.5 (9/24)	58.3 (14/24)	0.149
Gastroenterologist B			
Sensitivity (%)	57.1 (4/7)	71.4 (5/7)	0.500
Specificity (%)	52.9 (9/17)	76.5 (13/17)	0.140
Accuracy (%)	54.0 (13/24)	75.0 (18/24)	0.131
Gastroenterologist C			
Sensitivity (%)	14.3 (1/7)	71.4 (5/7)	0.051
Specificity (%)	35.3 (6/17)	76.5 (13/17)	0.018
Accuracy (%)	29.2 (7/24)	75.0 (18/24)	<0.001
All three gastroenterologists			
Sensitivity (%)	42.9 (9/21)	76.1 (16/21)	0.029
Specificity (%)	39.2 (20/51)	66.7 (34/51)	0.005
Accuracy (%)	40.3 (29/72)	69.4 (50/72)	<0.001

*CH-EUS* contrast-enhanced ultrasonography, *EUS* endoscopic ultrasonography.
